# Development of DArT markers and assessment of diversity in *Fusarium oxysporum* f. sp. *ciceris*, wilt pathogen of chickpea (*Cicer arietinum* L.)

**DOI:** 10.1186/1471-2164-15-454

**Published:** 2014-06-10

**Authors:** Mamta Sharma, Avuthu Nagavardhini, Mahendar Thudi, Raju Ghosh, Suresh Pande, Rajeev K Varshney

**Affiliations:** International Crops Research Institute for the Semi-Arid Tropics (ICRISAT), Patancheru, PO 502324 Andhra Pradesh India

**Keywords:** Chickpea, DArT, *Fusarium* wilt, Molecular markers, Races, Virulence

## Abstract

**Background:**

*Fusarium oxysporum* f. sp. *ciceris* (Foc), the causal agent of *Fusarium* wilt of chickpea is highly variable and frequent recurrence of virulent forms have affected chickpea production and exhausted valuable genetic resources. The severity and yield losses of *Fusarium* wilt differ from place to place owing to existence of physiological races among isolates. Diversity study of fungal population associated with a disease plays a major role in understanding and devising better disease control strategies. The advantages of using molecular markers to understand the distribution of genetic diversity in Foc populations is well understood. The recent development of Diversity Arrays Technology (DArT) offers new possibilities to study the diversity in pathogen population. In this study, we developed DArT markers for Foc population, analysed the genetic diversity existing within and among Foc isolates, compared the genotypic and phenotypic diversity and infer the race scenario of Foc in India.

**Results:**

We report the successful development of DArT markers for Foc and their utility in genotyping of Foc collections representing five chickpea growing agro-ecological zones of India. The DArT arrays revealed a total 1,813 polymorphic markers with an average genotyping call rate of 91.16% and a scoring reproducibility of 100%. Cluster analysis, principal coordinate analysis and population structure indicated that the different isolates of Foc were partially classified based on geographical source. Diversity in Foc population was compared with the phenotypic variability and it was found that DArT markers were able to group the isolates consistent with its virulence group. A number of race-specific unique and rare alleles were also detected.

**Conclusion:**

The present study generated significant information in terms of pathogenic and genetic diversity of Foc which could be used further for development and deployment of region-specific resistant cultivars of chickpea. The DArT markers were proved to be a powerful diagnostic tool to study the genotypic diversity in Foc. The high number of DArT markers allowed a greater resolution of genetic differences among isolates and enabled us to examine the extent of diversity in the Foc population present in India, as well as provided support to know the changing race scenario in Foc population.

**Electronic supplementary material:**

The online version of this article (doi: 10.1186/1471-2164-15-454) contains supplementary material, which is available to authorized users.

## Background

Chickpea (*Cicer arietinum* L.) or Garbanzo beans (Latin America) is the second largest cultivated legume crop after dry beans globally [1]. It is grown in 54 countries as a rainfed, post-rainy season and winter crop in subtropical South Asia, parts of Africa and Australia and as a spring season crop in the temperate and Mediterranean regions [1]. During 2012, chickpea covered a total of 12.14 million ha (M ha) area worldwide with a global production of 11.30 million tons (M t) and average productivity of 931.2 kg ha^−1^, where as in India it covered 8.32 M ha with the production of 7.58 M t and average yield of 912 kg ha^−1^[1]. India is the largest producer of chickpea and accounts for 68.47% of the total area and 67.02% of total production globally. Chickpea represents 35.16% of total pulse area and 50.34% of total pulse production in India [2].

Various biotic and abiotic stresses affect stable and high yields of chickpea crop worldwide. Among the biotic stresses, *Fusarium* wilt (FW), caused by the asexual, soil borne and seed borne fungus *Fusarium oxysporum* Schlecht and Emnd Snyd. & Hans. f. sp. *ciceris* (Padwick) Snyd. and Hans. (Foc), results in major economic losses ranging from 10-40% worldwide. It is estimated to cause 10-15% yield loss annually in India [3], but can result in 100% losses under favourable conditions. The pathogen enters host through roots and causes systemic infection by progressive vascular damage, wilting and finally death of the plant. The cultivation of resistant varieties is one of the most durable and economical practice for the management of FW. However, performance of varieties differs from place to place owing to existence of physiological races among the Foc isolates. Eight races of Foc (0, 1A, 1B/C, 2, 3, 4, 5 and 6) have been reported worldwide [4–6]. Races 1A (also known as race 1), 2, 3 and 4 have been reported from India, whereas races 0, 1B/C, 5 and 6 were found mainly in the Mediterranean region and in the United States (California). Among all the races, race 1 is more widespread and has been reported in India, California and the Mediterranean region. Pathogen also exhibit race specific differences in disease symptomatology; the yellowing and wilting [5], the first produces progressive yellowing of leaves and vascular discoloration and plant dies 40 days after inoculation of the pathogen, while later induces severe chlorosis, flaccidity and vascular discoloration and the plant dies 20 days after inoculation [7]. Races 0 and 1B/C induce yellowing symptoms, whereas the remaining races (1A, 2, 3, 4, 5 and 6) induce wilting. Recently, change in the race scenario of Foc has been reported by Dubey et al. [8] and Sharma et al. [9] in India. Therefore, the identification of pathogenic races of Foc is important for disease resistance breeding and for the efficient use of available FW resistant cultivars in chickpea.

Monitoring pathogenic variability of fungus based on DNA markers will greatly help in understanding pathogen diversity and their pathogenicity. In past, different kinds of molecular markers were employed to characterize Foc isolates, for instance, random amplified polymorphic DNA (RAPD) [10], restriction fragment length polymorphism (RFLP) [11], amplified fragment length polymorphism (AFLP) [12], inter simple sequence repeats (ISSR) [13] and simple sequence repeats (SSR) [13]. However, the marker systems are put to limited use because of lack of reliability and resolution (RAPD markers) or labour intensive and not amenable for throughput genotyping (AFLP, RFLP) or initial development costs are very high or require sequence information (SSRs). In this context, Diversity Arrays Technology (DArT) developed by Jaccoud et al. [14] is the best platform to generate thousands of markers in species like Foc with meagre genomic resources. This technology has been successfully employed in several crop plants for characterization of germplasm, trait mapping, and establishing marker trait associations [15–17].

The present study reports on the development of DArT markers and characterization of Foc isolates. The specific objectives of this study were i) to develop high density Foc DArT arrays ii) to assess effectiveness of DArT markers in diversity study on a set of 110 Foc isolates representing diverse agro-ecological zones of India and iii) Comparative assessment of genotypic and phenotypic data based on standard set of differential cultivars of chickpea. This new molecular marker system for Foc will serve as an additional resource to augment the existing systems to assist crop improvement efforts, disease management practices and genetic studies in chickpea.

## Results

### Virulence diversity

**Figure 1:**
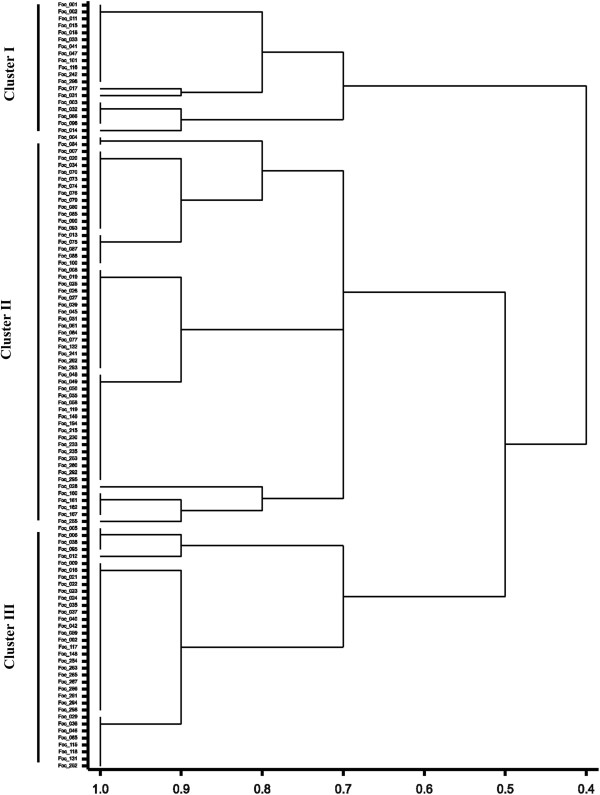
**Dendrogram derived from virulence analysis of 110 isolates of** ***F. oxysporum*** **f. sp**. ***ciceris*** **by UPGMA with 10 chickpea differential cultivars.**

Based on disease reaction of 110 isolates collected from five diverse chickpea growing agro-ecological zones like Central Zone (CZ), North East Plain Zone (NEPZ), North Hill Zone (NHZ), North West Plain Zone (NWPZ) and South zone (SZ) representing 13 states and 25 locations in India (Table 1) on a standard set of ten differentials, all the isolates showed a large variation in virulence (Additional file 1). The dendrogram based on virulence data on differentials grouped the isolates into three major Clusters (Figure 1). Grouping of the isolates clearly indicated the existence of more than one race in a region. Presence of race 1 was observed irrespective of zones in all the clusters. However, most of the isolates of race 1 reaction were grouped in Cluster I. Cluster II represented the isolates with least virulent reaction irrespective of location and Cluster III was dominated by race 6. The race reaction indicated the widespread occurrence of race 1 (46%) followed by new race 6 (32%) in India. Race 2 reactions were found only in 5 isolates (4.5%, Foc_014, Foc_017, Foc_018, Foc_015 and Foc_041) from Uttar Pradesh (UP), Maharashtra (MH) and Gujarat (GJ) and race 4 reactions in only one isolate from Bihar (BH) (Foc_011). None of the isolates used in the present study had a reaction similar to race 3. Sixteen isolates were found with different disease reaction which did not match to any race reactions. However, these isolates grouped together and their reaction was either susceptible or moderately resistant on JG 62, and resistant to other differential cultivars. Disease reactions of standard chickpea differential cultivars against different isolates of Foc are provided in Additional file 1.

**Table 1:** **Passport information of isolates used in** ***Fusarium oxysporum*** **f. sp** ***. ciceris*** **DArT array development, phenotyping and genotyping**

S. No.	Isolate ID	Location	State**	Agro-ecological zone***	Latitude	Longitude	Elevation (m)	Year of collection
1	Foc_001*	Patancheru	Andhra Pradesh	SZ	17°31'53" N	78°15'54" E	516	1995
2	Foc_002*	Patancheru	Andhra Pradesh	SZ	17°31'53" N	78°15'54" E	516	1995
3	Foc_003	Patancheru	Andhra Pradesh	SZ	17°31'53" N	78°15'54" E	516	1995
4	Foc_004*	Patancheru	Andhra Pradesh	SZ	17°31'53" N	78°15'54" E	516	2000
5	Foc_005*	Patancheru	Andhra Pradesh	SZ	17°31'53" N	78°15'54" E	516	2001
6	Foc_006*	Patancheru	Andhra Pradesh	SZ	17°31'53" N	78°15'54" E	516	2004
7	Foc_007*	Hisar	Haryana	NWPZ	29°10'00" N	75°43'00" E	202	2002
8	Foc_008	Hisar	Haryana	NWPZ	29°10'00" N	75°43'00" E	202	2003
9	Foc_009*	Hisar	Haryana	NWPZ	29°10'00" N	75°43'00" E	202	2004
10	Foc_011*	Dholi	Bihar	NEPZ	25°49' 00" N	72°34' 60”E	131	2005
11	Foc_012*	Dhaulakuan	Himachal Pradesh	NHZ	30°28'00" N	77°05'00" E	468	2005
12	Foc_013*	Gulbarga	Karnataka	SZ	17°19'59" N	76°49'59" E	458	2001
13	Foc_014*	Junagadh	Gujarat	CZ	21°31'00" N	70°28'00" E	119	2005
14	Foc_015*	Junagadh	Gujarat	CZ	21°31'00" N	70°28'00" E	119	2005
15	Foc_016*	Sehore	Madhya Pradesh	CZ	23°12'00" N	77°04'59" E	502	2005
16	Foc_017*	Badnapur	Maharashtra	CZ	19°52'00" N	75°43'60" E	498	2005
17	Foc_018*	Badnapur	Maharashtra	CZ	19°52'00" N	75°43'60" E	498	2005
18	Foc_019	Rahuri	Maharashtra	CZ	19°22'59" N	74°39'00" E	511	2004
19	Foc_020*	Rahuri	Maharashtra	CZ	19°22'59" N	74°39'00"E	511	2004
20	Foc_021*	Delhi	Delhi	NWPZ	28°40'00" N	77°13'00" E	213	2005
21	Foc_022*	Ludhiana	Punjab	NWPZ	30°54'00" N	75°51'00" E	243	2002
22	Foc_023*	Ludhiana	Punjab	NWPZ	30°54'00" N	75°51'00" E	243	2005
23	Foc_024	Gurdaspur	Punjab	NWPZ	32°03'00" N	75°27'00" E	241	2005
24	Foc_025*	Kanpur	Uttar Pradesh	NEPZ	26°28'00" N	80°21'00" E	128	2003
25	Foc_026*	Kanpur	Uttar Pradesh	NEPZ	26°28'00" N	80°21'00" E	128	2004
26	Foc_027	Kanpur	Uttar Pradesh	NEPZ	26°28'00" N	80°21'00" E	128	2004
27	Foc_028*	Pantnagar	Uttarakhand	NWPZ	29°20'04" N	79°28'25" E	344	2004
28	Foc_029*	Pantnagar	Uttarakhand	NWPZ	29°20'04" N	79°28'25"E	344	2004
29	Foc_031*	Kurnool	Andhra Pradesh	SZ	15°48'00" N	78°04'00" E	289	2006
30	Foc_032*	Kurnool	Andhra Pradesh	SZ	15°48'00" N	78°04'00" E	289	2006
31	Foc_033*	Akola	Maharashtra	CZ	20°43'59" N	77°00'00" E	283	2006
32	Foc_034*	Jabalpur	Madhya Pradesh	CZ	23°10'01" N	79°57'00" E	403	2006
33	Foc_035*	Jabalpur	Madhya Pradesh	CZ	23°10'01" N	79°57'00" E	403	2006
34	Foc_036*	Jabalpur	Madhya Pradesh	CZ	23°10'01" N	79°57'00" E	403	2006
35	Foc_037*	Jabalpur	Madhya Pradesh	CZ	23°10'01" N	79°57'00" E	403	2006
36	Foc_038*	Patancheru	Andhra Pradesh	SZ	17°31'53" N	78°15'54" E	516	2005
37	Foc_039*	Dharwad	Karnataka	SZ	15°28'00" N	75°01'00" E	700	2007
38	Foc_040	Patancheru	Andhra Pradesh	SZ	17°31'53" N	78°15'54" E	516	1980
39	Foc_041	Kanpur	Uttar Pradesh	NEPZ	26°28'00" N	80°21'00" E	128	1980
40	Foc_042	Gurdaspur	Punjab	NWPZ	32°03'00" N	75°27'00" E	241	1980
41	Foc_045	Delhi	Delhi	NWPZ	28°40'00" N	77°13'00" E	213	2007
42	Foc_046*	Delhi	Delhi	NWPZ	28°40'00" N	77°13'00" E	213	2007
43	Foc_047*	Delhi	Delhi	NWPZ	28°40'00" N	77°13'00" E	213	2007
44	Foc_048*	Delhi	Delhi	NWPZ	28°40'00" N	77°13'00" E	213	2007
45	Foc_049	Delhi	Delhi	NWPZ	28°40'00" N	77°13'00" E	213	2007
46	Foc_050	Dharwad	Karnataka	SZ	15°28'00" N	75°01'00" E	700	2007
47	Foc_051	Dharwad	Karnataka	SZ	15°28'00" N	75°01'00" E	700	2007
48	Foc_055	Pantnagar	Uttarakhand	NWPZ	29°20'04" N	79°28'25" E	344	2007
49	Foc_058	Dhaulakuan	Himachal Pradesh	NHZ	30°28'00" N	77°05'00" E	468	2008
50	Foc_059*	Dhaulakuan	Himachal Pradesh	NHZ	30°28'00" N	77°05'00" E	468	2008
51	Foc_061	Dhaulakuan	Himachal Pradesh	NHZ	30°28'00" N	77°05'00" E	468	2008
52	Foc_064*	Gurdaspur	Punjab	NWPZ	32°03'00" N	75°27'00" E	241	2008
53	Foc_065	Hisar	Haryana	NWPZ	29°10'00" N	75°43'00" E	202	2008
54	Foc_066*	Patancheru	Andhra Pradesh	SZ	17°31'53" N	78°15'54" E	516	2008
55	Foc_070*	Patancheru	Andhra Pradesh	SZ	17°31'53" N	78°15'54" E	516	2008
56	Foc_073*	Patancheru	Andhra Pradesh	SZ	17°31'53" N	78°15'54" E	516	2008
57	Foc_074*	Patancheru	Andhra Pradesh	SZ	17°31'53" N	78°15'54" E	516	2008
58	Foc_075*	Patancheru	Andhra Pradesh	SZ	17°31'53" N	78°15'54" E	516	2008
59	Foc_076*	Patancheru	Andhra Pradesh	SZ	17°31'53" N	78°15'54" E	516	2008
60	Foc_077	Dhaulakuan	Himachal Pradesh	NHZ	30°28'00" N	77°05'00" E	468	2008
61	Foc_079	Dhaulakuan	Himachal Pradesh	NHZ	30°28'00" N	77°05'00" E	468	2008
62	Foc_080	Patancheru	Andhra Pradesh	SZ	17°31'53" N	78°15'54" E	516	2009
63	Foc_084	Patancheru	Andhra Pradesh	SZ	17°31'53" N	78°15'54" E	516	2009
64	Foc_085	Patancheru	Andhra Pradesh	SZ	17°31'53" N	78°15'54" E	516	2009
65	Foc_087	Patancheru	Andhra Pradesh	SZ	17°31'53" N	78°15'54" E	516	2009
66	Foc_088*	Patancheru	Andhra Pradesh	SZ	17°31'53" N	78°15'54" E	516	2009
67	Foc_090*	Patancheru	Andhra Pradesh	SZ	17°31'53" N	78°15'54" E	516	2009
68	Foc_092	Patancheru	Andhra Pradesh	SZ	17°31'53" N	78°15'54" E	516	2009
69	Foc_093*	Patancheru	Andhra Pradesh	SZ	17°31'53" N	78°15'54" E	516	2009
70	Foc_095	Patancheru	Andhra Pradesh	SZ	17°31'53" N	78°15'54" E	516	2009
71	Foc_096	Patancheru	Andhra Pradesh	SZ	17°31'53" N	78°15'54" E	516	2009
72	Foc_100	Patancheru	Andhra Pradesh	SZ	17°31'53" N	78°15'54" E	516	2009
73	Foc_101	Patancheru	Andhra Pradesh	SZ	17°31'53" N	78°15'54" E	516	2009
74	Foc_115*	Satna	Madhya Pradesh	CZ	24°34'59" N	80°49'59" E	318	2009
75	Foc_116*	Satna	Madhya Pradesh	CZ	24°34'59" N	80°49'59" E	318	2009
76	Foc_117*	Satna	Madhya Pradesh	CZ	24°34'59" N	80°49'59" E	318	2009
77	Foc_118*	Satna	Madhya Pradesh	CZ	24°34'59" N	80°49'59" E	318	2009
78	Foc_119	Damoh	Madhya Pradesh	CZ	23°49'59" N	79°27'00" E	354	2009
79	Foc_131*	Rewa	Madhya Pradesh	CZ	24°31'59" N	81°18'00" E	275	2009
80	Foc_132*	Satna	Madhya Pradesh	CZ	24°34'59" N	80°49'59" E	318	2009
81	Foc_146	Katni	Madhya Pradesh	CZ	23°47'00" N	80° 27'00" E	392	2009
82	Foc_148*	Kanpur	Uttar Pradesh	NEPZ	26°28'00" N	80°21'00" E	128	2009
83	Foc_160	Dharwad	Karnataka	SZ	15°28'00" N	75°01'00" E	700	2010
84	Foc_161	Dharwad	Karnataka	SZ	15°28'00" N	75°01'00" E	700	2010
85	Foc_162	Dharwad	Karnataka	SZ	15°28'00" N	75°01'00" E	700	2010
86	Foc_167	Dharwad	Karnataka	SZ	15°28'00" N	75°01'00" E	700	2010
87	Foc_194*	Rajnandgaon	Chhattisgarh	CZ	21°06'00" N	81°02'00" E	330	2010
88	Foc_215*	Durg	Chhattisgarh	CZ	21°10'59" N	81°16'59" E	288	2010
89	Foc_230	Durg	Chhattisgarh	CZ	21°10'59" N	81°16'59" E	288	2010
90	Foc_233*	Durg	Chhattisgarh	CZ	21°10'59" N	81°16'59" E	288	2010
91	Foc_235*	Kabirdham	Chhattisgarh	CZ	22°01'00" N	81°15'00" E	345	2010
92	Foc_241*	Sehore	Madhya Pradesh	CZ	23°12'00" N	77°04'59" E	502	2010
93	Foc_242	Patancheru	Andhra Pradesh	SZ	17°31'53" N	78°15'54" E	516	2010
94	Foc_252*	Kanpur	Uttar Pradesh	NEPZ	26°28'00" N	80°21'00" E	128	2010
95	Foc_253	Kanpur	Uttar Pradesh	NEPZ	26°28'00" N	80°21'00" E	128	2010
96	Foc_254	Kanpur	Uttar Pradesh	NEPZ	26°28'00" N	80°21'00" E	128	2010
97	Foc_255	Kanpur	Uttar Pradesh	NEPZ	26°28'00" N	80°21'00" E	128	2010
98	Foc_260	Kanpur	Uttar Pradesh	NEPZ	26°28'00" N	80°21'00" E	128	2010
99	Foc_262	Jabalpur	Madhya Pradesh	CZ	23°10'01" N	79°57'00" E	403	2010
100	Foc_263*	Jabalpur	Madhya Pradesh	CZ	23°10'01" N	79°57'00" E	403	2010
101	Foc_265	Jabalpur	Madhya Pradesh	CZ	23°10'01" N	79°57'00" E	403	2010
102	Foc_267	Jabalpur	Madhya Pradesh	CZ	23°10'01" N	79°57'00" E	403	2010
103	Foc_286	Jabalpur	Madhya Pradesh	CZ	23°10'01" N	79°57'00" E	403	2010
104	Foc_291*	Dhaulakaun	Himachal Pradesh	NHZ	30°28'00" N	77°05'00" E	468	2010
105	Foc_292	Kotabag	Uttarakhand	NWPZ	29°23'60" N	79°17'60" E	655	2010
106	Foc_293	Junagadh	Gujarat	CZ	21°31'00" N	70°28'00" E	119	2010
107	Foc_294	Junagadh	Gujarat	CZ	21°31'00" N	70°28'00" E	119	2010
108	Foc_295	Patancheru	Andhra Pradesh	SZ	17°31'53" N	78°15'54" E	516	2009
109	Foc_296	Kanpur	Uttar Pradesh	NEPZ	26°28'00" N	80°21'00" E	128	2010
110	Foc_298	Hisar	Haryana	NWPZ	29°10'00" N	75°43'00" E	202	2004

*Isolates used for construction of DArT array.

**AP - Andhra Pradesh, BR - Bihar, CG - Chhattisgarh, DL - Delhi, GJ - Gujarat, HR - Haryana, HP - Himachal Pradesh, KA - Karnataka, MP - Madhya Pradesh, MH - Maharashtra, PB - Punjab, UP - Uttar Pradesh, UK - Uttarakhand,

***CZ - Central zone, NEPZ - North east plain zone, NHZ - North hill zone, NWPZ - North west plain zone and SZ - South zone.

### DArT array development and evaluation

**Figure 2:**
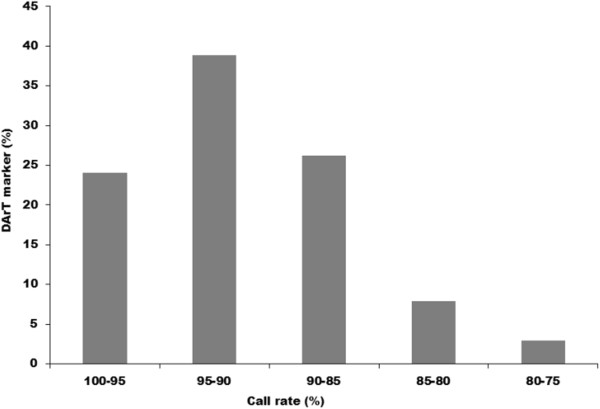
**Distribution of call rate among the 1,813 DArT markers.**

A total of 61 Foc isolates that represent whole genome diversity of Foc were used for developing the diversity arrays using *Pst*I/*Hpa*II restriction endonucleases. A total of 4,991 DArT markers were developed from *Pst*I/*Hpa*II restriction endonucleases combinations generated from a mixture of DNA of 61 Foc isolates. Of 4,991 markers, 1,813 markers were found polymorphic using DArTsoft 7.3. The overall marker quality was high, with approximately > 90% call rate (Figure 2). The call rate of the polymorphic markers ranged from 75-100% with an average of 91.16% and had scoring reproducibility 100%. The allele frequencies of polymorphic DArT markers ranged from 0.50-0.99 (mean = 0.87 ± 0.08).

**Figure 3:**
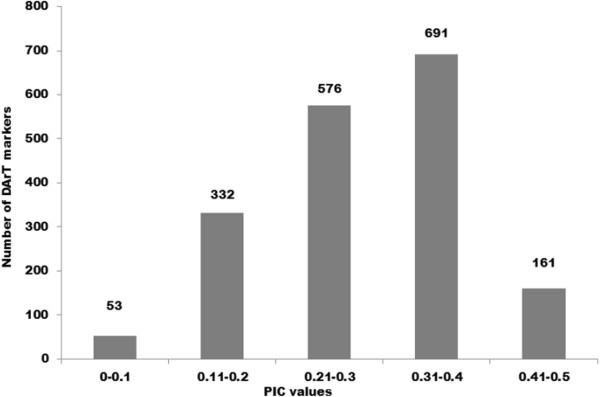
**Number of DArT markers in different PIC value classes.**

Average polymorphism information content (PIC) value for DArT markers was 0.28. About 2.92% of markers had PIC value in the range of 0.00-0.10, while 18.31% and 31.77% markers were in the range of 0.11-0.20 and 0.21-0.30, respectively (Figure 3). Further, 38.11% and 8.88% of markers had the PIC values in a range of 0.31-0.40 and 0.41-0.50 respectively. Overall the distribution of PIC values was asymmetrical and skewed towards the higher value (Figure 3). Further, when the quality of the DArT markers was analyzed against their performance, determined by the call rate and PIC values, 97.07% of the polymorphic DArT markers (n = 1760) were in the 80-100% quality category with an average PIC value of 0.28 and a call rate of 91.55% (Additional file 2). The average PIC value increased with the average quality value. The PIC values for 14 markers possessing marker quality of <50% ranged from 0.04-0.30. Of 438 markers with a quality of more than 80%, the 358 markers had a PIC value of >0.30. Out of 1,813 polymorphic DArT markers in Foc analyzed, there are 691 and 161 markers in the PIC value range of 0.30-0.40 and 0.40-0.50, respectively (Figure 2).

### Genetic relationship among Foc isolates

**Figure 4:**
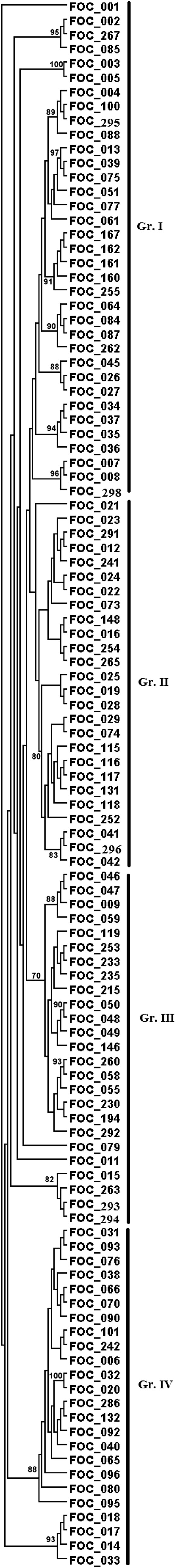
**Cluster analysis of** ***F. oxysporum*** **f. sp**. ***ciceris*** **isolates based on 1,813 polymorphic DArT markers.**

A total of 110 isolates of Foc (Table 1) were employed for gaining insights into the genetic diversity patterns. Using the 1,813 polymorphic markers, UPGMA dendrogram was generated with the 110 isolates (Figure 4). The estimated reliability of the branches of the dendrogram was also supported by the bootstrap values as shown in Figure 4. All the isolates tested could be divided into four major clusters with 29, 26, 20, 19 isolates clustered in Cluster I, Cluster II, Cluster IV and Cluster III respectively. However, a total of 16 isolates were grouped separately from the above mentioned major clusters. In each cluster, isolates from different agro-ecological zones was distributed. However, certain region specific sub-grouping within each cluster was found.

**Figure 5:**
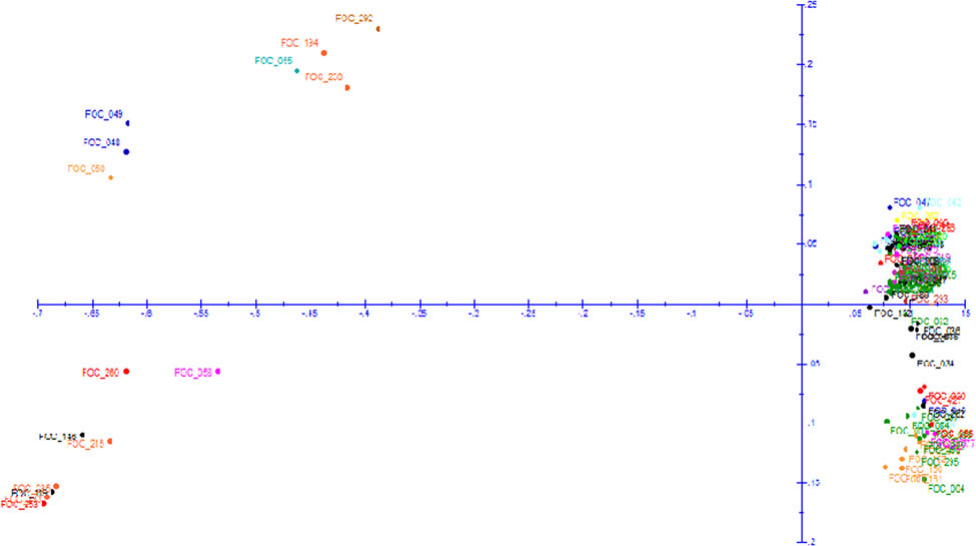
**Distribution of 110** ***F. oxysporum*** **f. sp**. ***ciceris*** **isolates in different quadrants based principal coordinate analysis using 1,813 DArT markers.** The principal coordinate I explain 70.04% and principal coordinate II explain 8.33% variation.

Cluster I represented 49% isolates from SZ (AP and KA) and 34% from NWPZ and NEPZ (UP, HR and PB). In the second Cluster, 58% isolates were from NWPZ and NHZ (HP, DL, HR, and UP) and 34% from CZ (MP and MH). The third Cluster was smallest with the isolates mostly from NWPZ and NEPZ (58%, DL, HR, HP, UP and UK) and 38% from CZ (CG). Fourth cluster represented 67% isolates from SZ (AP) and remaining from CZ and NWPZ (MP, MH and GJ). Few isolates (Foc_001, Foc_002, Foc_003, Foc_005, Foc_085, Foc_267) from AP grouped separately and clustered at the top of the dendrogram. Also few isolates were found to be isolated from major groups for instance isolates from BR (Foc_011), HP (Foc_079), GJ (Foc_015, 293, 294) and MP (Foc_263).The PCoA based on polymorphic DArT marker data derived from 110 isolates, confirmed the four different groups found by UPGMA dendrogram (Figure 5). The two dimensions of PCoA explained 70.04% and 8.33% of the variation present in the genetic distance calculated between isolates. These two components were responsible for a total 78.37% of the variation observed among the isolates. The right-hand groups (in upper and lower quadrates) proved to be clustered around the PCoA X- axis with isolates originating them different agro-ecological zone. This group corresponded to the isolates from Cluster III of the UPGMA dendrogram. The left hand groups were spread widely along both the PCoA X- axis and PCoA Y- axis. These groups represented the isolates from Cluster-I, II and IV of the UPGMA dendrogram.

### Population structure of Foc

**Figure 6:**
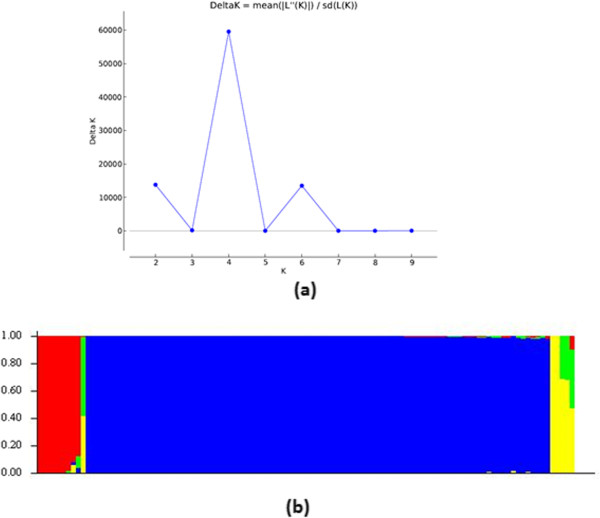
**Population structure among 110** ***F. oxysporum*** **f. sp**. ***ciceris*** **isolates (a) The highest delta K value is observed for K value 4, indicating 4 sub-populations among the Foc isolates used in the study (b) Genetic composition of different sub-populations.**

In order to assess the population structure and number of sub-populations, 1,813 polymorphic DArT markers were used on 110 Foc isolates employing STRUCTURE 2.3.4. Based on the maximum likelihood and delta K (ΔK) values, four sub-populations were determined in 110 Foc isolates (Figure 6). Each vertical bar in the figure represents a single Foc isolate and its inferred proportion of genetic admixture. The parts with different colours represent four different sub-populations. Using a membership probability threshold of 0.60, 9 isolates were assigned to sub-population 1, 1 isolate to sub-population 2, 95 isolates to sub-population 3 and 5 isolates to sub-population 4. Detailed information of sub-populations and respective isolate within each sub-population is provided in Additional file 3. Sub-population 1, 2 and 4 have genetic admixture, however sub-population 3 have homogeneous genetic background.

### Allele frequency

The allelic composition revealed the predominance of common (959) and most frequent alleles (852) in the total 1,813 polymorphic alleles (Additional file 4). Foc population contained a lower proportion of rare alleles. Among 1,813 polymorphic markers, most frequent alleles were represented by 46.9% and common alleles contributed to 52.8%. However, 48 unique alleles were detected in the Foc isolates whose reaction did not match with any specific races. Further, race specific unique alleles were also identified for different race specific isolates for instance 52 (race 1), 4 (race 4) and 33 (race 6).

## Discussion

The importance of understanding genetic diversity in any fungal species collection is critical for their effective management. Although tremendous progress has been made in terms of availability of molecular markers to study the genetic diversity in Foc [7, 8, 10, 12]; availability of sound, reliable and cost-effective marker platform is still lacking. In the present study, we present the first complete report on the development of DArT marker platform for any fungal pathogen. To the best of our knowledge, this is the first attempt to develop DArT markers for Foc and investigation on the utility of these markers in genetic diversity of large number of Foc isolates collected from various regions in India. We further demonstrated the pathogenic races, as determined by the disease reaction on differential cultivars-correlated to inferred DArT genotyping.

The DArT technology combines a reduction of genome complexity with high throughput and cost-effective hybridization based polymorphism detection. The advantage of using these DArT markers is that they can simultaneously type several thousand loci in a single array and provide a cost-effective and sequence independent tool for whole genome fingerprinting [14]. The DArT technology has been applied to a number of plants and animals [16, 18–20]. However, no information is available on any plant pathogen till date. In this study, the new DArT platform for Foc was found acceptable and provided robust information about the genetic diversity in this collection. Out of 4,991 DArT markers, 1,813 markers were found to be useful in providing a complete picture of genetic diversity in the Foc collection of 110 isolates. Overall, the average PIC value for DArT marker was found to be lower than that observed in other plant species (compared with plant species due to the non-availability of information on DArT markers on pathogens), where similar markers were developed [21, 22], but comparable to that observed in pigeonpea [17] and sugar beet [23]. Nearly nine per cent of DArT markers have a PIC value in the range of 0.41-0.50 and these markers therefore may be considered informative.

DArT markers developed in this study effectively detected diversity among Foc isolates. All the three types of DArT diversity analysis (cluster analysis, PCoA and population structure) indicated that the different isolates were successfully classified by the marker system and reflected high genetic diversity. The phylogeny of the Foc isolates inferred from DArT markers by cluster analysis indicated 4 major Clusters among them. The subgroup clustering pattern with in each major Cluster was consistent, indicating that the DArT markers faithfully detected small differences among isolates. A similar finding was reported earlier from different markers systems like RAPD [7, 8], AFLP [12] and SSR [8] with considerably less resolution and less transferability.

Virulence analysis on a set of differential cultivars is required to authenticate the groups generated by the molecular markers. In this study, virulence analysis based on standard set of differentials showed the existence of highly variable populations of Foc in different agro-climatic zones in India. The reactions on differential cultivars of chickpea indicated the existence of races 1, 2, 4 and 6 in India. In addition few isolates (13.63%) did not show match with any of the race reaction and was grouped separately in both the dendrograms. Previous studies based on differentials reported the presence of eight pathogenic races (races 0, 1A, 1B/C, 2, 3, 4, 5 and 6) in Foc based on disease reactions on a set of differential chickpea cultivars [4, 24].

Race 1 represented 47% of total Foc isolates, of which most of the isolates grouped in Cluster I. Previous studies reported race 1 only from AP and KA states of India [4], however present diversity based on DArT indicated its presence in all the agro-ecological zones of India, with predominance in SZ. The high genetic diversity among the race 1 isolates of Foc revealed its diverse geographical inhabitation under the present scenario. This widespread distribution of race 1 can be attributed to the seed transmission through large exchange of germplasm that took place more in last one decade. Among all the races, race 1 is more widespread and has been reported in India, California, and the Mediterranean region [24].

Race 6 represented 29% of total Foc isolates, of which most of the isolates were grouped into Cluster II. The race 6 was previously confined to Mediterranean basin. It was first reported from California [6] later reported from Spain [25], Israel and Morocco [26]. Occurrence of this new race of Foc in India mainly appeared to be a recent introduction through seed transmission and pathogen genetic variability from accumulation of mutations over the time. According to Jimenez et al. (7), there may have little or no selection for resistance –breaking races of Foc, minimizing the number of parallel events in acquisition of virulence, which are frequently observed in other system. Thus, unlike other pathosystems (e.g. downy mildew, rust and blast) there may have been little or no selection for resistance breaking races of Foc, which minimizes the probabilities of obtaining parallel changes in virulence. They further stated that it is not probable that the stepwise evolution of races in Foc resulted from the selection by specific resistance in chickpea populations, as widespread use of race 1 A-resistant cultivars in India has not yet led to reports on development of race 6. On the contrary, we speculate that stepwise evolution of races in Foc resulted from selection by specific resistance in chickpea populations or repeated migration has occurred rather than the independent evolution of races in different regions or as expected from an ancient lineage race 1 could be the common ancestor of all races. Presence of race 6 in India is also indicated by Dubey et al. [8]. Further, there are reports that races 1 and 6 have similar virulence pattern on chickpea differentials [27] and high genetic similarities [28]. Earlier researchers indicated that it is possible that misclassification of these isolates occurred during pathogenicity test. It is also possible that these two pathogenic races were not yet evolutionarily separated from each other to have developed diagnostic and separate pathogenicity test.

The isolates grouped in Cluster III represent those isolates which had susceptible reaction with less virulence on JG 62 cultivar and resistance reaction on all the other differential cultivars. This group corresponded to 19% of total Foc isolates representing NZ. Difference in virulence among Foc isolates originating from southern and northern partes of India has also been reported by Dubey et al. [8]. The distribution of virulence within and among populations of Foc is probably the result of different selection pressure exerted by a specific resistant gene in the chickpea varieties cultivated in the area. This is in agreement with the observations by Lebeda and Petrzelova [29] in the case of *Bremia lactucae*. Also, this could be the result of shift in the cultivation of chickpea from northern India to southern India [30], which further supported the low frequency of race 2, originally reported from Kanpur, north India. Race 3 disease reaction was not found in any of the isolates. The absence of race 3 in the present collection is in agreement with Gurjar et al. [31]. They found completely different reaction of race 3 and reported its resemblance to *Fusarium proliferatum* based on phylogenetic analysis.

The isolates in Cluster IV mainly represent multiple race reaction race 1, race 2, race 4 and race 6. Regardless of the origin, the similarities of race composition between the different zones of India indicated that repeated migration of infected seeds.

The unique, common and most frequent alleles among the total 1,813 polymorphic markers reported in the present study are novel features. Such features, to the best of our knowledge, have not been reported in other diversity study. Race specific alleles detected in the present study will be very useful for molecular profiling of a particular race. Further, occurrence of common alleles will be useful for understanding the similarity and also molecular profiling of Foc isolates from diverse geographical locations. Two rare alleles observed specific to UP location could be useful in the detection of location specific Foc isolates.

## Conclusions

The present study generated significant information in terms of pathogenic and genetic diversity of Foc which could be used further for deployment of region-specific resistant varieties of chickpea. The study clearly highlighted the occurrence of multiple/new races of Foc in India and suggests the continuous monitoring of changes in Foc population to check the susceptibility of widely grown resistant cultivars against these races. The DArT markers developed in the present study are powerful diagnostic tools for understanding the diversity in Foc population in chickpea. In this study, we reported the successful development of DArT marker platform for Foc and their utility in genotyping of Foc collections. This DArT marker system complemented the microsatellite markers developed previously for Foc. The high number of DArT markers allowed a greater resolution of genetic differences among isolates and enabled us to examine the extent of variability in the Foc population present in India, as well as provided support to know the changing race scenario in Foc population. Further, the DArT markers identified in this study could be used to study the distribution of these Foc races, and to facilitate the efficient deployment of available host resistance. These markers should assist in the early detection of introduced race(s), as well as of changes in the relative frequencies of different races that might occur in response to the use of resistant chickpea cultivars.

The DArT arrays developed for Foc in this study are available with the Center of Excellence in Genomics (CEG) at ICRISAT. Also the Foc isolates used in the present study are available with Legumes Pathology Unit at ICRISAT for further use by any scientific community.

## Methods

### Fungal culture

A total 110 Foc isolates collected from 25 locations representing 13 states and five agro-ecological zones of India (NEPZ, NWPZ, NHZ, CZ and SZ) were used in the present study (Table 1). For developing DArT arrays, 61 isolates were chosen representing all the zones and races in India. All the isolates were purified and monoconidial cultures were maintained on potato dextrose agar (PDA) slants at 4°C. The pathogenicity of all the isolates was proved following root-dip inoculation method under controlled environmental conditions [32].

### Virulence diversity

The virulence diversity among 110 representative isolates of Foc (Table 1) was studied on a set of 10 standard chickpea differential cultivars, namely JG 62, JG 74, Chaffa, WR 315, L 550, BG 212, CPS 1, C 104, Annigeri 1, and K 850 [4] under greenhouse conditions following the root dip inoculation method. Monoconidial cultures of each Foc isolate was multiplied separately in 100 ml potato-dextrose broth (PDB) in 250 ml flasks and incubated in a rotary shaker at 120 rpm for seven days at 25 ± 1°C with 12-h photoperiod of fluorescent light. After seven days, the entire content of each flask was homogenized separately and diluted with sterilized distilled water to a final inoculum concentration of 5 × 10^5^ conidia ml^−1^. Seeds of all the 10 chickpea differential cultivars were surface sterilized separately using 2% sodium hypochlorite for 2 min, rinsed in sterile water and germinated for eight days in sterilized sand filled in plastic bags. The eight-day-old seedlings of each cultivar were carefully uprooted and roots were washed under running water to remove excess sand. The root dipping for all the cultivars was done using each isolate separately. Root tips around 0.5 cm long were cut off to facilitate the entry of the pathogen into the roots. The roots of the seedlings were then dipped in the Foc inoculum for 1–2 min to enable conidia to adhere to the roots. The inoculated seedlings were transplanted into a pre-irrigated sterile vertisol and sand (3:1) mixture and incubated at 25 ± 2°C in the glasshouse. Three pots with five seedlings were used for each isolate and experiment was repeated twice. Un-inoculated seedlings of each cultivar were maintained as control. The wilt incidence was recorded regularly up to crop maturity. The disease reactions was graded as resistant (0–20% wilt), moderately susceptible (>20–50% wilt), and susceptible (>50% wilt). The race classification of isolates was done based on their reaction on a set of differential cultivars [4, 25]. The reactions of genotypes that could be scored unequivocally as susceptible/moderately susceptible were designated as 1 and resistant as 0 for analysis. Binary matrices were analyzed by GENSTAT (Edition 14).

### DNA extraction

DNA extraction was done by following cetyltrimethylammonium bromide (CTAB) method [33]. All the 110 isolates were grown in PDB 250 ml flasks and incubated in a rotary shaker at 120 rpm at 25 ± 1°C for five days. In brief, mycelia were harvested by filtering through mira cloth, and washed repeatedly with sterile distilled water to remove excess of salts adhering to it. One gram mycelium was crushed in liquid nitrogen and transferred into 7.5 ml pre-warmed (65°C) DNA extraction buffer [1 M Tris–HCl (pH 8.0), 5 M NaCl, 0.5 M ethylene diamine tetra acetic acid (EDTA; pH 8.0) and 2% CTAB], mixed well and incubated in a water bath at 65°C with gentle shaking for 45 min. Equal volume of chloroform: isoamyl alcohol (24:1 v/v) was added and mixed gently to denature proteins and centrifuged at 12,857 g for 10 min. DNA was precipitated with 0.6 volume of chilled ethanol and 0.1 volume of 3 M sodium acetate (pH 5.2) and centrifuged at 18,514 g for 15 min. The pellets were washed twice with chilled 70% ethanol, dried at room temperature, re-suspended in 100 μl sterile TE (10 mM Tris–HCl buffer and 1 mM EDTA; pH 8) and stored at −20°C. Isolated DNA was run in 0.8% agarose gels to check the quality and quantity of DNA. DNA concentration in each isolate was adjusted to ca. 100 ng μl^−1^.

### Development of DArT arrays

#### Selection of restriction enzymes

A set of 61 Foc isolates that represent the diversity in the Foc isolates selected based on geographical distribution and prevalence of races in India were used for developing the DArT arrays (Table 1). For complexity reduction a set 12 restriction endonucleases (*Ban*II, *Apo*I, *Alu*I, *Bst*I, *Hpa*II, *Taq*I, *Mse*I, *Sty*I, *Sac*I, *Rsa*II, *Acl*I and *Apa*I) were tested to determine the restriction endonuclease combination that provided the best complexity reduction. Genomic representations were prepared using *Pst*I*/Hpa*II combination. Approximately 100 ng genomic DNA was digested with *Pst*I*/Hpa*II combination and the resulting fragments ligated to a *Pst*I adapter (Adapter 1: 5’CACGATGGATCCAGTGCA3’ and Adapter 2: 5’CTGGATCC ATCGTGCA3’) with T4 DNA ligase (NEB, UK). Primer annealing to this adapter was used in PCR to amplify complexity reduced representation of a sample with the following cycling parameters: 94°C for 4 min, followed by 30 cycles of 94°C for 20 seconds, 58°C for 40 seconds, 72°C for 1 min, and final extension at 72°C for 7 min.

#### DArT library preparation

Library was prepared by ligating the pooled genomic representations into the PCR 2.1-TOPO vector using the TOPO cloning kit (Invitrogen, USA) and transforming these into an electrocompetent *Eschercia coli* strain (TOP 10) according to the manufacturer’s instructions. Transformants were selected on a medium containing ampicillin and X-gal and grown in 384-well plates containing Luria broth medium supplemented with 100 mg/ml ampicillin and a freezing mix. The cloned representation fragments were amplified in a 15 μl reaction containing 0.1 M each of forward and reverse M13 primers (F: 5’ GTTTTCCCAGTCACGACGTTG 3’and R: 5’ TGAGCGGATAACAATTTCACA CAG 3’) and 0.3U of *Taq* polymerase (Sibenzyme Ltd., Russia) using the following PCR cycling conditions: 95°C for 3 min, 57°C for 35 seconds, 72°C for 1 min, followed by 40 cycles of 94°C for 35 seconds, 52°C for 35 seconds, and 72°C for 1 min. The approximate size of amplified fragments ranged from 200 to 1,000 bp. After amplification, the PCR products were precipitated with one volume of isopropanol at room temperature and washed once with 100 μl of 77% ethanol. The ethanol was then removed and the products dried as per Jaccoud et al. [14]. The DNA pellet was re-suspended in spotting buffer (1 M sucrose + 50% DMSO) and the resulting solutions of clean amplified DNA fragments from 4,994 clones were used in printing a replicated array. A total of 4,994 fragments were spotted on to poly-L-lysine coated slides (Erie Scientific Co., USA) using a Micro-GridII arrayer (Biorobotics, UK) in replication with approximately 10% missing spots. After printing, slides were left at room temperature for at least one day and then processed by heating them in hot water at 92°C for 2 min and drying by centrifugation.

#### Genotyping

Genomic representations of all 110 Foc isolates were generated using the same complexity reduction method (*Pst*I/*Hpa*II), which has been developed for DArT library. Representations were precipitated with one volume of isopropanol, denatured at 95°C for 3 min, and labelled with fluorescent dye (1.5 μl of 500 μl Cy3/Cy5-dUTP, random decamers synthesized by Sigma, Australia) using the exo-Klenow fragment of *E. coli* DNA polymerase I (NEB, UK). For hybridization, labelled representations (also called targets) were mixed with 50 μl of DArT hybridizer (50 : 5 : 1 mixture of Express Hyb buffer (Clontech, USA), 10gL^−1^ herring sperm DNA (Sigma-Aldrich, USA), and the 6-FAM-labeled poly-linker fragment of the plasmid that was used for library preparation, and denatured at 95°C for 3 min [14]. After denaturing labelled targets were hybridized onto the microarray surface and covered with a glass coverslip (24 × 9 × 60 mm, Menzel-Glazer, Germany). Slides were quickly placed into a 65°C water bath for overnight hybridization. After overnight hybridization at 65°C, the cover slips were removed, slides were placed into slide-racks, and washed in 4 steps: step 1:19X SSC 0.1% SDS for 5 min; step 2: in 19X SSC for 5 min; step 3: in 0.29X SSC for 2 min; and step 4: in 0.029X SSC for 30 seconds. Slides were centrifuged and dried in vacuum desiccators.

#### Image analysis and polymorphism scoring

After the slides were dry, they were scanned using a conFocal laser scanner (Tecan LS 300 scanner, Mannedorf, Switzerland) and images were generated for each of the fluorescent dyes using appropriate laser/filter combinations at appropriate wavelengths (Cy3: 543 nm, Cy5: 633 nm, 6-FAM: 488 nm). DArTsoft 7.3 (developed by DArT P/L, Australia), was used to automatically analyse each batch of microarray TIF image pairs generated in the experiment. The relative hybridization intensity of each clone on each slide was determined by dividing the hybridization signal in the target channel (genomic representation) by the hybridization signal in the reference channel (polylinker). The software localized the spots, rejected those with a weak reference signal, and the relative hybridization intensities of polymorphic clones in the representation hybridized to a slide were converted into present “1” or absent “0” scores based on the membership probability estimates computed by the clustering algorithm.

#### Data analysis

DArTsoft 7.3 computed several quality parameters such as, (i) between-cluster variance in relative hybridization intensity as a percentage of the total variance (*P* value), (ii) the percentage of data points with defined allele calls (call rate), (iii) the polymorphism information content (PIC), a measure of informativeness of these DArT markers. The PIC value was calculated according to Xia et al*.*[34].

#### Unique and rare alleles

Occurrence of unique, rare, common and most frequent alleles was also calculated. Unique alleles are those that are present in one isolate or one group of isolates belonging to same race but absent in other isolates or races. Rare alleles are those whose frequency is ≤ 1% in the investigating materials. Common alleles are those occurring between 1-20% in the investigated materials while those occurring with >20% classified as most frequent alleles.

#### Diversity analysis

The 0/1 binary matrix of the markers was used for the calculation of genetic similarity. The bootstrapped Unweighted Pair Group Method with Algorithmic Mean (UPGMA) Dendrogram was generated with 1,000 replication using PAUP 4.0 and Dendroscope. Principal coordinate analysis (PCoA) for genetic similarity matrix was obtained on Jaccard’s similarity coefficient to display the position of the isolates in a two-dimensional space.

#### STRUCTURE analysis

Model-based clustering, employing a Bayesian algorithm, was applied to infer the genetic structure of the 110 Foc isolates using STRUCTURE software version 2.3.4 [35]. The program was run assuming a population admixture model and correlated allele frequencies. The number of assumed groups (K) was set to vary between 1 and 10, and for each value of K three times independently MCMC (Markov Chain Monte Carlo) of 50,000 iterations was run in order to verify that the estimates were consistent across runs, of which the first 10,000 were discarded as burn-in. The value of K with the highest likelihood can be interpreted to correspond to an estimate for the underlying number of groups. The admixture model provided the membership probabilities of each genotype to each group inferred.

## Electronic supplementary material

Additional file 1: **Disease reaction of standard chickpea differential cultivars against different isolates of** ***Fusarium oxysporum*** **f. sp**. ***ciceris*** **in different agro-ecological zones.** (DOCX 46 KB)

Additional file 2: **Features of 1,813 polymorphic DArT loci based on marker analysis in 110** ***Fusarium oxysporum*** **f. sp**. ***ciceris*** **isolates.** (XLSX 73 KB)

Additional file 3: **Population structure of 110 Foc isolates indicating sub-population and Foc isolate numbers.** (TIFF 873 KB)

Additional file 4: **Allele frequency of 1,813 polymorphic DArT loci in 110 isolates of** ***Fusarium oxysporum*** **f. sp**. ***ciceris.*** (XLSX 715 KB)
