# Supplementary material for: Development of DArT markers and assessment of diversity in Fusarium oxysporum f. sp. ciceris, wilt pathogen of chickpea (Cicer arietinum L.)
Source: BMC Genomics. 2014 Jun 10;15(1):454. doi: 10.1186/1471-2164-15-454 (PMC4070567; doi:10.1186/1471-2164-15-454)
Supplement: Supplementary file 1 — Additional file 1: Disease reaction of standard chickpea differential cultivars against different isolates of Fusarium oxysporum f. sp. ciceris in different agro-ecological zones. (DOCX 46 KB) [file 12864_2014_6145_MOESM1_ESM.docx]

**Additional File 1. Disease reaction of standard chickpea differential cultivars against different isolates of *Fusarium oxysporum* f. sp. *ciceris***

| **State/Location** | **Isolate** | **C-104** | **JG62** | **JG-74** | **CPS-1** | **BG-212** | **WR-315** | **Annegiri-1** | **Chaffa** | **L-550** | **K-850** | **Race** |
| --- | --- | --- | --- | --- | --- | --- | --- | --- | --- | --- | --- | --- |
| **Andhra Pradesh (SZ)** | |  |  |  |  |  |  |  |  |  |  |  |
| Patancheru | Foc_001 | S | S | R | R | R | R | M | S | S | M | Race 1 |
| Patancheru | Foc_002 | S | S | R | R | R | R | M | S | S | M | Race 1 |
| Patancheru | Foc_003 | S | S | R | R | R | R | S | S | S | M | Race 1 |
| Patancheru | Foc_004 | R | S | R | R | R | R | S | S | M | R | Race 1 |
| Patancheru | Foc_005 | S | S | R | R | R | R | S | S | R | R | Race 1 |
| Patancheru | Foc_006 | S | S | R | R | R | R | S | S | M | M | Race 1 |
| Patancheru | Foc_038 | S | S | R | R | R | R | S | S | M | M | Race 1 |
| Patancheru | Foc_040 | S | S | R | R | R | R | R | M | M | R | Race 1 |
| Patancheru | Foc_066 | S | S | R | R | R | R | S | S | S | M | Race 1 |
| Patancheru | Foc_070 | S | S | R | R | R | R | M | S | M | M | Race 1 |
| Patancheru | Foc_073 | S | S | R | R | R | R | R | S | M | R | Race 6 |
| Patancheru | Foc_074 | S | S | R | R | R | R | M | S | R | R | Race 6 |
| Patancheru | Foc_075 | S | S | R | R | R | R | M | S | M | R | Race 1 |
| Patancheru | Foc_076 | S | S | R | R | R | R | R | S | M | M | Race 1 |
| Patancheru | Foc_080 | S | S | R | R | R | R | M | S | M | M | Race 1 |
| Patancheru | Foc_084 | R | S | R | R | R | R | S | S | M | M | Race 1 |
| Patancheru | Foc_085 | S | S | R | R | R | R | M | S | M | M | Race 1 |
| Patancheru | Foc_087 | M | S | R | R | R | R | M | S | M | M | Race 1 |
| Patancheru | Foc_088 | M | S | R | R | R | R | M | S | M | M | Race 1 |
| Patancheru | Foc_090 | S | S | R | R | R | R | M | S | M | M | Race 1 |
| Patancheru | Foc_092 | S | S | R | R | R | R | R | M | M | R | Race 1 |
| Patancheru | Foc_093 | S | S | R | R | R | R | M | S | M | M | Race 1 |
| Patancheru | Foc_095 | S | S | R | R | R | R | S | S | M | M | Race 1 |
| Patancheru | Foc_096 | S | S | R | R | R | R | S | S | S | M | Race 1 |
| Patancheru | Foc_100 | R | S | R | R | R | R | M | S | R | M | Race 1 |
| Patancheru | Foc_101 | S | S | R | R | R | R | M | S | S | M | Race 1 |
| Patancheru | Foc_242 | S | S | R | R | R | R | M | S | S | R | Race 1 |
| Patancheru | Foc_295 | R | R | R | R | R | R | R | R | R | R | Race 1 |
| Kurnool | Foc_031 | S | S | R | R | R | R | M | S | S | S | Race 1 |
| Kurnool | Foc_032 | S | S | M | R | R | R | S | S | S | M | Race 1 |
| **Bihar (NEPZ)** |  |  |  |  |  |  |  |  |  |  |  |  |
| Dholi | Foc_011 | S | S | R | M | R | R | R | S | S | R | Race 4 |
| **Chhattisgarh (CZ)** | |  |  |  |  |  |  |  |  |  |  |  |
| Rajnandgaon | Foc_194 | R | R | R | R | R | R | R | R | R | R | New Reaction |
| Durg | Foc_215 | R | M | R | R | R | R | R | R | R | R | New Reaction |
| Durg | Foc_230 | R | M | R | R | R | R | R | R | R | R | New Reaction |
| Durg | Foc_233 | R | M | R | R | R | R | R | R | R | R | New Reaction |
| Kabirdham | Foc_235 | R | R | R | R | R | R | R | R | R | R | New Reaction |
| **Delhi (NWPZ)** |  |  |  |  |  |  |  |  |  |  |  |  |
| Delhi | Foc_021 | S | S | R | R | R | R | R | M | R | R | Race 6 |
| Delhi | Foc_045 | M | S | R | M | R | R | R | R | M | R | Race 6 |
| Delhi | Foc_046 | S | S | R | R | R | R | R | R | S | R | Race 6 |
| Delhi | Foc_047 | S | S | R | R | R | R | R | S | S | R | Race 6 |
| Delhi | Foc_048 | R | M | R | R | R | R | R | R | R | R | New Reaction |
| Delhi | Foc_049 | R | R | R | R | R | R | R | R | R | R | New Reaction |
| **Gujarat (CZ)** |  |  |  |  |  |  |  |  |  |  |  |  |
| Junagadh | Foc_014 | S | S | M | M | M | R | S | S | S | S | Race 2 |
| Junagadh | Foc_015 | S | S | M | M | M | R | M | S | S | M | Race 2 |
| Junagadh | Foc_293 | M | S | R | R | R | R | R | M | M | M | Race 6 |
| Junagadh | Foc_294 | S | S | R | R | R | R | R | M | M | M | Race 6 |
| **Haryana (NWPZ)** | |  |  |  |  |  |  |  |  |  |  |  |
| Hisar | Foc_007 | S | S | R | R | R | R | R | S | M | R | Race 1 |
| Hisar | Foc_008 | R | S | R | R | R | R | R | R | R | R | Race 1 |
| Hisar | Foc_009 | S | S | R | R | R | R | R | R | M | R | Race 6 |
| Hisar | Foc_065 | S | S | R | R | M | R | R | M | S | M | Race 1 |
| Hisar | Foc_298 | S | S | R | R | R | R | R | R | R | R | Race 6 |
| **Himachal Pradesh (NHZ)** | |  |  |  |  |  |  |  |  |  |  |  |
| Dhaulakaun | Foc_012 | S | S | R | R | R | R | S | M | M | M | Race 1 |
| Dhaulakaun | Foc_058 | R | R | R | R | R | R | R | R | R | R | New Reaction |
| Dhaulakaun | Foc_059 | S | S | R | R | R | R | R | R | M | R | Race 6 |
| Dhaulakaun | Foc_061 | R | S | R | R | R | R | R | R | R | R | Race 1 |
| Dhaulakaun | Foc_077 | R | S | R | R | R | R | R | R | R | R | Race 1 |
| Dhaulakaun | Foc_079 | S | S | R | R | R | R | R | S | M | M | Race 6 |
| Dhaulakaun | Foc_291 | S | S | R | R | R | R | R | R | M | M | Race 1 |
| **Karnataka (SZ)** |  |  |  |  |  |  |  |  |  |  |  |  |
| Gulbarga | Foc_013 | R | S | R | R | R | R | R | S | M | M | Race 1 |
| Dharwad | Foc_039 | R | S | R | R | R | R | R | M | M | M | Race 1 |
| Dharwad | Foc_050 | R | R | R | R | R | R | R | R | R | R | New Reaction |
| Dharwad | Foc_051 | R | S | R | R | R | R | R | R | R | R | Race 1 |
| Dharwad | Foc_160 | M | S | R | R | R | R | M | S | R | S | Race 1 |
| Dharwad | Foc_161 | M | S | R | R | R | R | R | S | R | S | Race 1 |
| Dharwad | Foc_162 | M | S | R | R | R | R | R | S | R | S | Race 1 |
| Dharwad | Foc_167 | M | S | R | R | R | R | R | S | R | S | Race 1 |
| **Madhya Pradesh (CZ)** | |  |  |  |  |  |  |  |  |  |  |  |
| Sehore | Foc_016 | S | S | R | R | R | R | R | M | M | R | Race 6 |
| Sehore | Foc_241 | R | S | R | R | R | R | R | R | R | R | Race 1 |
| Jabalpur | Foc_034 | S | S | R | R | R | R | M | S | M | M | Race 1 |
| Jabalpur | Foc_035 | S | S | R | R | R | R | R | R | R | R | Race 1 |
| Jabalpur | Foc_036 | S | S | R | R | R | R | R | R | S | R | Race 6 |
| Jabalpur | Foc_037 | S | S | R | R | R | R | M | R | M | M | Race 1 |
| Jabalpur | Foc_262 | R | S | R | R | R | R | R | R | R | R | Race 1 |
| Jabalpur | Foc_263 | S | S | R | R | R | R | R | R | M | R | Race 6 |
| Jabalpur | Foc_265 | S | S | R | R | R | R | R | R | R | R | Race 6 |
| Jabalpur | Foc_267 | S | S | R | R | R | R | R | R | R | R | Race 1 |
| Jabalpur | Foc_286 | S | S | R | R | R | R | R | R | M | R | Race 6 |
| Satna | Foc_115 | S | S | R | R | R | R | R | M | S | R | Race 6 |
| Satna | Foc_116 | S | S | M | R | R | R | M | S | S | M | Race 6 |
| Satna | Foc_117 | S | S | R | R | R | R | R | M | M | M | Race 6 |
| Satna | Foc_118 | S | S | M | R | R | R | M | M | S | R | Race 6 |
| Satna | Foc_132 | R | S | M | R | R | R | R | R | R | R | Race 6 |
| Damoh | Foc_119 | R | M | R | R | R | R | R | R | R | R | New Reaction |
| Rewa | Foc_131 | S | S | M | R | R | R | R | M | S | R | Race 6 |
| Katni | Foc_146 | R | R | R | R | R | R | R | R | R | R | New Reaction |
| **Maharashtra (CZ)** | |  |  |  |  |  |  |  |  |  |  |  |
| Badnapur | Foc_017 | S | S | M | S | M | R | M | S | S | S | Race 2 |
| Badnapur | Foc_018 | S | S | M | R | M | R | R | S | S | M | Race 2 |
| Rahuri | Foc_019 | R | S | R | R | R | R | R | R | M | R | Race 6 |
| Rahuri | Foc_020 | S | S | R | R | R | R | M | S | M | M | Race 1 |
| Akola | Foc_033 | S | S | R | R | R | R | R | S | S | R | Race 1 |
| **Punjab (NWPZ)** | |  |  |  |  |  |  |  |  |  |  |  |
| Ludhiana | Foc_022 | S | S | R | R | R | R | R | M | M | R | Race 6 |
| Ludhiana | Foc_023 | S | S | R | R | R | R | R | M | M | R | Race 6 |
| Gurdaspur | Foc_024 | S | S | R | R | R | R | R | R | M | R | Race 6 |
| Gurdaspur | Foc_042 | S | S | R | R | R | R | R | M | M | R | Race 6 |
| Gurdaspur | Foc_064 | M | S | R | R | R | R | R | M | M | R | Race 6 |
| **Uttar Pradesh (NEPZ)** | |  |  |  |  |  |  |  |  |  |  |  |
| Kanpur | Foc_025 | M | S | R | R | R | R | R | M | M | R | Race 6 |
| Kanpur | Foc_026 | M | S | R | R | R | R | R | R | R | R | Race 6 |
| Kanpur | Foc_027 | M | S | R | R | R | R | R | R | M | M | Race 6 |
| Kanpur | Foc_041 | S | S | M | R | M | R | R | S | S | R | Race 2 |
| Kanpur | Foc_148 | S | S | R | R | R | R | R | R | R | R | Race 6 |
| Kanpur | Foc_252 | S | S | R | R | R | R | R | R | S | R | Race 6 |
| Kanpur | Foc_253 | R | R | R | R | R | R | R | R | R | R | New Reaction |
| Kanpur | Foc_254 | S | S | R | R | R | R | R | R | M | R | Race 6 |
| Kanpur | Foc_255 | M | M | R | R | R | R | M | S | R | S | Race 1 |
| Kanpur | Foc_260 | R | R | R | R | R | R | R | R | R | R | New Reaction |
| Kanpur | Foc_296 | S | S | R | R | M | R | R | S | S | M | Race 6 |
| **Uttarakhand (NWPZ)** | |  |  |  |  |  |  |  |  |  |  |  |
| Pantnagar | Foc_028 | M | S | R | R | R | R | M | M | M | S | Race 1 |
| Pantnagar | Foc_029 | S | S | M | R | R | R | R | M | S | R | Race 1 |
| Pantnagar | Foc_055 | R | R | R | R | R | R | R | R | R | R | New Reaction |
| Kotabag | Foc_292 | R | M | R | R | R | R | R | R | R | R | New Reaction |

R = Resistant (0-20% wilt), M = Moderately susceptible (21-50% wilt) and S = Susceptible (≥51% wilt).

NEPZ = North eastern plane zone, NWPZ = North western plane zone, NHZ = North hill zone, CZ = Central zone and SZ = South zone.
