# Supplementary figures and images for: Development of DArT markers and assessment of diversity in Fusarium oxysporum f. sp. ciceris, wilt pathogen of chickpea (Cicer arietinum L.)
Source: BMC Genomics. 2014 Jun 10;15(1):454. doi: 10.1186/1471-2164-15-454 (PMC4070567; doi:10.1186/1471-2164-15-454)

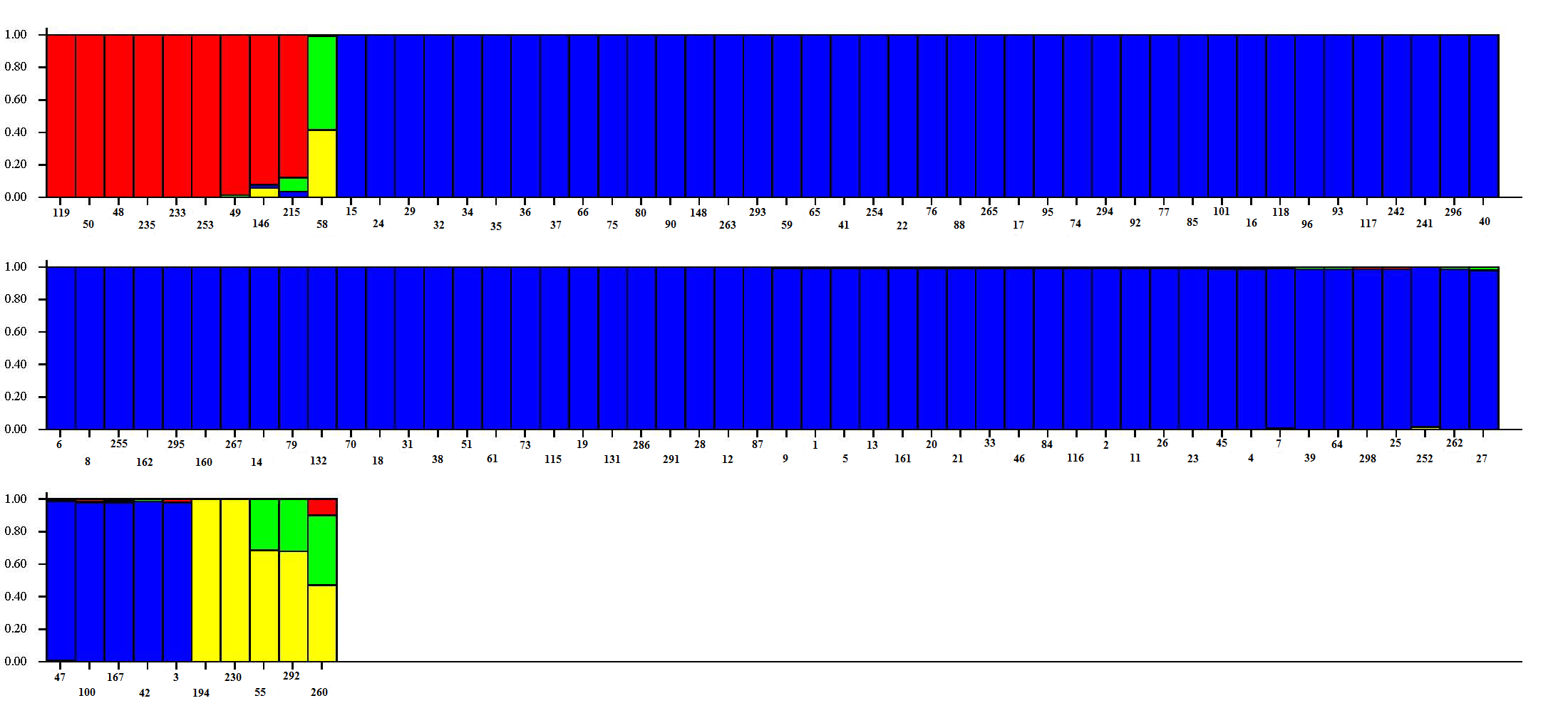

Supplement: Supplementary file 3 — Additional file 3: Population structure of 110 Foc isolates indicating sub-population and Foc isolate numbers. (TIFF 873 KB) [file 12864_2014_6145_MOESM3_ESM.tiff]
